# Effects of cytochrome P450 (CYP3A4 and CYP2C19) inhibition and induction on the exposure of selumetinib, a MEK1/2 inhibitor, in healthy subjects: results from two clinical trials

**DOI:** 10.1007/s00228-016-2153-7

**Published:** 2016-11-26

**Authors:** Angela W. Dymond, Karen So, Paul Martin, Yifan Huang, Paul Severin, David Mathews, Eleanor Lisbon, Gabriella Mariani

**Affiliations:** 1 AstraZeneca, Alderley Park, Macclesfield, Cheshire, SK10 4TG UK; 2 AstraZeneca R&D, AstraZeneca Global Medicines Development, Da Vinci Building, Melbourn, Royston, Hertfordshire SB8 6HB UK; 3 AstraZeneca, Gaithersburg, MD 20878 USA; 4 Covance Laboratories, Inc., Madison, WI 53704 USA; 5 Quintiles Phase I Services, Overland Park, KS, 66211 USA

**Keywords:** Selumetinib, Exposure, Cytochrome P450, Inhibition, Induction, Pharmacokinetic profile

## Abstract

**Purpose:**

Two phase I, open-label trials in healthy subjects assessed whether co-administration with CYP3A4/CYP2C19 inhibitors, itraconazole/fluconazole (study A), or CYP3A4 inducer, rifampicin (study B), affects the exposure, safety/tolerability and pharmacokinetics of selumetinib and its metabolite *N*-desmethyl selumetinib.

**Methods:**

In study A (*n* = 26), subjects received a single dose of selumetinib 25 mg and, after 4 days of washout, were randomized to treatment 1 (itraconazole 200 mg twice daily on days 1–11) or treatment 2 (fluconazole 400 mg on day 1 then 200 mg/day on days 2–11) plus co-administration of single-dose selumetinib 25 mg on day 8 (selumetinib staggered 4 h after itraconazole/fluconazole dose); Twenty-one days after discharge/washout, subjects received the alternate treatment. In study B (*n* = 22), subjects received a single dose of selumetinib 75 mg (day 1) then rifampicin 600 mg/day (days 4–14) plus a single dose of selumetinib 75 mg on day 12. Pharmacokinetic analysis and safety assessments were performed.

**Results:**

Selumetinib co-administered with itraconazole, fluconazole (selumetinib staggered 4 h after itraconazole/fluconazole dose), or rifampicin was well tolerated. Selumetinib exposure was higher when co-administered with itraconazole or fluconazole (area under the plasma concentration-time curve (AUC) increased by 49 and 53%, respectively; maximum plasma concentration (*C*_max_) increased by 19 and 26%, respectively) but lower when co-dosed with rifampicin (AUC and *C*_max_ decreased by 51 and 26%, respectively) versus selumetinib dosed alone. Co-administration with itraconazole or rifampicin decreased *N*-desmethyl selumetinib AUC_(0–t)_ (11 and 55%, respectively), and *C*_max_ (25 and 18%, respectively), with fluconazole, AUC_(0–t)_ increased by 40%, but there was no effect on *C*_max_.

**Conclusions:**

Co-administration of CYP3A4/CYP2C19 inhibitors will likely increase exposure to selumetinib, while CYP3A4 inducers will likely reduce its exposure.

**Electronic supplementary material:**

The online version of this article (doi:10.1007/s00228-016-2153-7) contains supplementary material, which is available to authorized users.

## Introduction

Selumetinib (AZD6244, ARRY-142886) is an oral, potent and selective, allosteric MEK1/2 inhibitor [1] in clinical development for a variety of different tumor types, including a phase III study in differentiated thyroid cancer [2] (NCT01843062) and a phase II registration study in neurofibromatosis type 1 [3] (NCT01362803).

In vitro data from reaction phenotyping studies have shown that selumetinib can undergo metabolism by phase I (mainly oxidation through cytochrome P450 (CYP) enzymes) and phase II (conjugation with glucuronides) metabolic pathways (unpublished data^1^). CYP3A4 is the pre-dominant isoform responsible for selumetinib oxidative metabolism with CYP1A2, CYP2C9, CYP2C19, CYP2E1, and CYP3A5 that is also involved to a smaller extent. CYP3A4, in particular, is involved in the metabolism of a wide range of compounds, including various drugs and toxins [4–6]. Exposure of selumetinib may be influenced by CYP inhibitors or inducers, in particular by inhibitors or inducers of CYP3A4 or CYP2C19, potentially affecting the safety profile or efficacy of selumetinib. Potent CYP3A4 and CYP2C19 modulators include itraconazole and fluconazole (antifungal agents) and rifampicin (an antibiotic agent); these agents can be used as representative inhibitors or inducers of these CYP enzymes. Itraconazole and fluconazole are not totally selective towards CYP3A4 and CYP2C19, respectively, but demonstrate the most potent inhibition of these CYPs. Rifampicin induces many CYPs but exerts its most potent induction on CYP3A4. Since selumetinib will be given to patients who are potentially taking concurrent therapies, which could potentially modify the activity of these enzymes, assessing the exposure of selumetinib when taken together with such agents is extremely important for evaluating the effects on the safety profile and/or efficacy of selumetinib.

Here, we report two clinical trials in healthy subjects assessing the exposure of selumetinib and its metabolite: *N*-desmethyl selumetinib when co-administered with the CYP3A4 or CYP2C19 inhibitors, itraconazole or fluconazole, respectively, or the CYP3A4 inducer, rifampicin.

## Subjects and methods

Both phase I open-label, single-center trials in healthy subjects were performed by Quintiles Phase I Services (Overland Park, KS, USA). The trials were conducted in accordance with the ethical principles outlined in the Declaration of Helsinki and the International Conference on Harmonization Good Clinical Practice. Written informed consent was obtained from all subjects prior to any study-related procedures.

The primary study objectives were to investigate the effects of the potent CYP3A4 or CYP2C19 inhibitors, itraconazole 200 mg and fluconazole 200 mg (400 mg on first day dose) at multiple doses, respectively, on the exposure of a single oral dose of selumetinib 25 mg (NCT02093728), and the effects of CYP3A4 induction from multiple 600 mg oral doses of rifampicin on the exposure of a single oral dose of selumetinib 75 mg (NCT02046850). Secondary objectives included assessment of the pharmacokinetics (PKs) of *N*-desmethyl selumetinib following co-dosing of selumetinib with itraconazole, fluconazole, or rifampicin, and the safety and tolerability of selumetinib.

### Subjects

For both studies, healthy male and female (of nonchildbearing potential) subjects aged 18–45 years, weighing 50–100 kg, and with a body mass index of 18–30 kg/m^2^ were eligible for enrollment. Additional information regarding the inclusion and exclusion criteria is included as Online Resource 1.

### Study design and experimental treatments

A sample size of 20 evaluable healthy subjects was determined for each trial based on the desire to gain adequate information while exposing as few healthy subjects as possible to study procedures. As demonstrated in the expected 90% confidence intervals (CIs) for the geometric least squares mean ratios for area under the plasma concentration-time curve (AUC) and maximum plasma concentration (*C*_max_), this sample size would provide an adequate precision for the estimation of potential drug-drug interaction effects. The expected 90% CIs were calculated using historical data. The within-subject estimates of the coefficient of variation (CV) were derived from the relative bioavailability trial (NCT01635023) [7] of 16.9% for AUC and 34.0% for *C*_max_ for selumetinib (manuscript in preparation).

Twenty-six and 24 healthy subjects were planned for enrollment into the itraconazole/fluconazole (study A) and rifampicin (study B) studies, respectively, in order to ensure at least 20 subjects completed each study, assuming an approximate dropout rate of 20%.

Selumetinib 75 mg is the intended therapeutic dose and the maximum dose permitted in Western healthy subjects. A lower dose of selumetinib, 25 mg, was used in the itraconazole/fluconazole trial to limit any increased exposure of selumetinib resulting from potential drug-drug interactions, to below the normal exposure at 75 mg. In both trials, the formulation of selumetinib used was identical to that in the ongoing phase III studies (oral capsules, containing 25 mg freebase, equivalent of selumetinib Hyd-Sulfate; Patheon, Cincinnati, USA).

#### Itraconazole/fluconazole trial (study A)

**Fig. 1:**
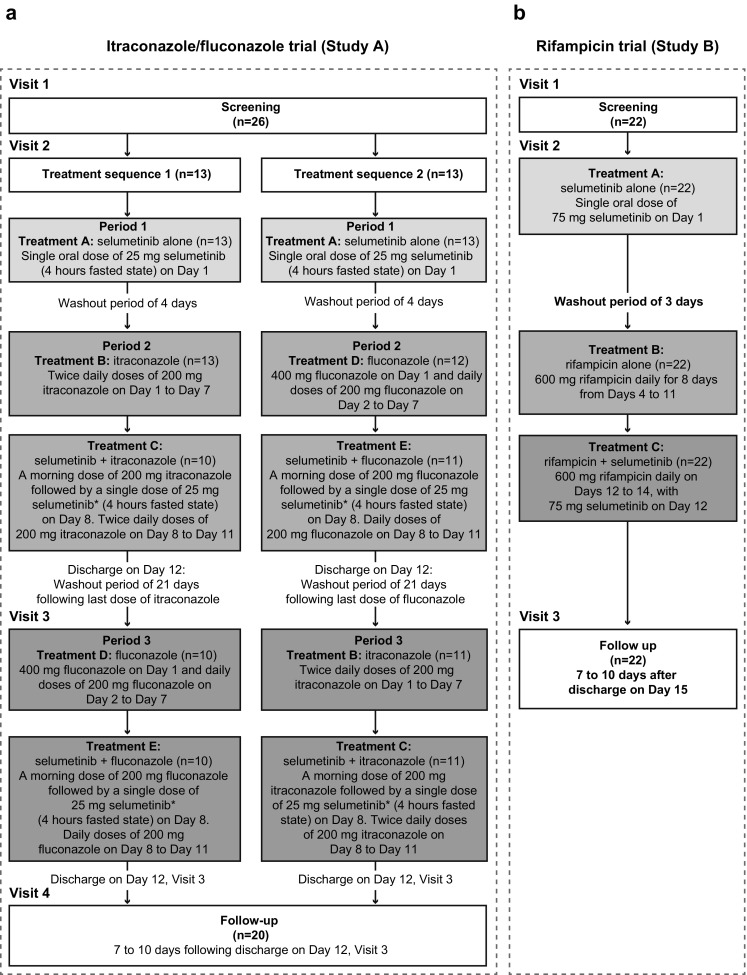
Design of study A (itraconazole and fluconazole (**a**)) and study B (rifampicin (**b**)). *Asterisk* indicates that selumetinib staggered 4 h after itraconazole/fluconazole dose

The phase I, open-label, single-center, double-crossover-sequence, partially randomized (for assignment to treatment sequence), three-period itraconazole/fluconazole trial consisted of four visits to the study center (Fig. 1a). Subjects were screened during visit 1, which is 28 days prior to visit 2.

On day 1 of visit 2, subjects were randomized to one of two treatment sequences and admitted for residence at the study center up to day 12; standardized meals were provided during residency. During dosing period 1, all subjects received a single dose of selumetinib 25 mg followed by a 4-day washout period. In dosing period 2, during days 1–11, subjects received either itraconazole (200 mg twice daily) or fluconazole (a 400 mg loading dose on day 1 followed by 200 mg once daily thereafter); all subjects received a second dose of selumetinib 25 mg on day 8. Safety assessments were performed, and blood samples were collected for PK analysis up until day 12. Subjects were discharged on day 12 and completed a washout period of at least 21 days.

During visit 3, dosing period 3 comprised days 1–11 when subjects received the alternate treatment regimen (fluconazole 400 mg on day 1 followed by 200 mg once daily thereafter or itraconazole 200 mg twice daily) in a crossover fashion. All subjects received a second dose of selumetinib of 25 mg on day 8. Subjects were discharged on day 12. A follow-up visit, visit 4, was scheduled for 7–10 days after discharge from the study center.

Dose regimens for itraconazole and fluconazole were selected following consideration of the product Summary of Product Characteristics (SPCs) [8, 9] and internal expertise with the aim of maximizing the inhibitory effects on CYP3A4 and CYP2C19 while limiting exposure to the agents as appropriate. Subjects were administered with accepted clinical regimens of itraconazole (200 mg twice daily) or fluconazole (a 400 mg loading dose and 200 mg daily thereafter), respectively, for 7 days prior to selumetinib. Continued dosing of itraconazole or fluconazole for a further 3–4 days after selumetinib dosing ensured that the maximum enzyme inhibitory effects were maintained during the selumetinib sampling period. Selumetinib was administered 4 h after a light breakfast (approximately 4 h after the itraconazole or fluconazole dose), and subjects continued to fast at least 4 h post-dose. Fluids were not allowed from 1 h pre- until 1 h post-dose, with the exception of 240 mL water to swallow the capsules. Fluconazole was dosed with a light breakfast; there were no restrictions regarding dosing with food in the SPC [8]. Itraconazole was dosed with a light breakfast and dinner as per SPC guidelines to ensure complete absorption [9].

#### Rifampicin trial (study B)

The rifampicin open-label, fixed-sequence, single-center trial consisted of three visits to the study center (Fig. 1b). Subjects were screened during visit 1, which is 28 days prior to visit 2. During visit 2, subjects received a single oral dose of selumetinib 75 mg (3 × 25 mg capsules) on day 1 and resided at the study center up to day 15. On day 4, daily oral rifampicin 600 mg (capsule) was commenced and continued up to day 14, with a further single dose of selumetinib 75 mg co-administered (at the same time) on day 12. Safety assessments were performed, and blood samples were collected for PK analysis up until day 15. Subjects were discharged from the study center on day 15. The final visit, visit 3, was for follow-up and took place 7–10 days after discharge.

Subjects received daily dosing of rifampicin 600 mg for 8 days to maximize the induction effect on CYP3A4 [10]. Selumetinib 75 mg was administered in a fasted state; subjects were maintained in a fasted state overnight, for a minimum of 10 h until 4 h post-dose. Similarly, subjects were required to fast for at least 10 h prior to rifampicin administration and remained in the fasted state for a further 1 h post-dose. Fluids were not allowed from 1 h pre- until 1 h post-dose, with the exception of water needed to swallow investigational products.

### Pharmacokinetic assessments

Serial blood samples (2 mL) to measure plasma selumetinib PK in the rifampicin trial were collected pre-selumetinib administration (0 h) and at 0.5, 1.0, 1.5, 2.0, 2.5, 3.0, 4.0, 5.0, 6.0, 8.0, 12.0, 24.0, 36.0, 48.0, and 72.0 h post-dose on days 1–4 and 12–15. The itraconazole/fluconazole trial included additional sampling time points, at 3.5 and 96.0 h post-selumetinib administration on days 1–5 of period 1 and days 8–12 of periods 2 and 3. Blood samples were collected prior to itraconazole or fluconazole administration on days 6 to 8 to determine trough itraconazole or fluconazole concentrations; rifampicin concentrations were measured at 2 h post-dose. In study B, the 4β-hydroxycholesterol to cholesterol concentration ratio was calculated as a biomarker of CYP3A4 induction. Blood samples (4 mL) to measure 4β-hydroxycholesterol and cholesterol prior to administration of rifampicin or selumetinib were collected on day 12 and before rifampicin administration on days 4 and 14. Samples were analyzed by Covance on behalf of AstraZeneca R&D, using an appropriate bioanalytical method [e.g., 11, 12]. Additional information regarding the pharmacokinetic assessments, including bioanalytical methodology and assay performance, is included as Online Resource 2.

### Safety and tolerability

Adverse events (reported by system organ class and preferred term using the Medical Dictionary for Regulatory Activities (MedDRA) version 17.0), vital signs, 12-lead electrocardiograms (ECGs), and clinical laboratory tests were recorded to assess safety and tolerability in both trials. Adverse events were collected from day 1, period 1 of visit 2, until the follow-up visit in each trial. Serious adverse events were collected from the time of informed consent until follow-up. Each adverse event was assigned to the period of study treatment in which it started or worsened. Additional information regarding safety and tolerability is included as Online Resource 3.

## Results

### Trial populations

Twenty-six and 22 healthy subjects were enrolled in the itraconazole/fluconazole and rifampicin studies, respectively (Online Resource 4 illustrates the passage of subjects through the studies). Subjects in both trials had an age range of 18–44 years, and the majority were male (>90%) and of similar mean body mass index (26 kg/m^2^). Approximately half of all subjects in the itraconazole/fluconazole and rifampicin trials were White (50.0 and 45.5%, respectively) and half were Black/African Americans (50 and 45.5%, respectively); in the rifampicin trial, one subject (4.5%) was classed as American Indian/Alaska Native and one subject (4.5%) was classed as “Other.”

In the itraconazole/fluconazole trial, all 26 subjects were randomized to one of two treatment sequences and all received study treatment. Twenty-one subjects (80.8%) completed all treatment sequences, and 20 (76.9%) completed the study (one subject was lost to follow-up). Five subjects discontinued partway through the study; three subjects (11.5%) had protocol deviations (two subjects receiving selumetinib/itraconazole in period 2; one subject receiving selumetinib/fluconazole in period 2), one (3.8%) violated the study site rules (the subject left and returned to the premises unescorted and unannounced during residential stay in period 2, fluconazole pre-dosing), and one withdrew consent (during period 2 selumetinib/itraconazole co-administration). In the rifampicin trial, all 22 (100%) subjects received single-sequence treatment and completed the study.

### Pharmacokinetic results

#### Selumetinib co-dosed with itraconazole

**Fig. 2:**
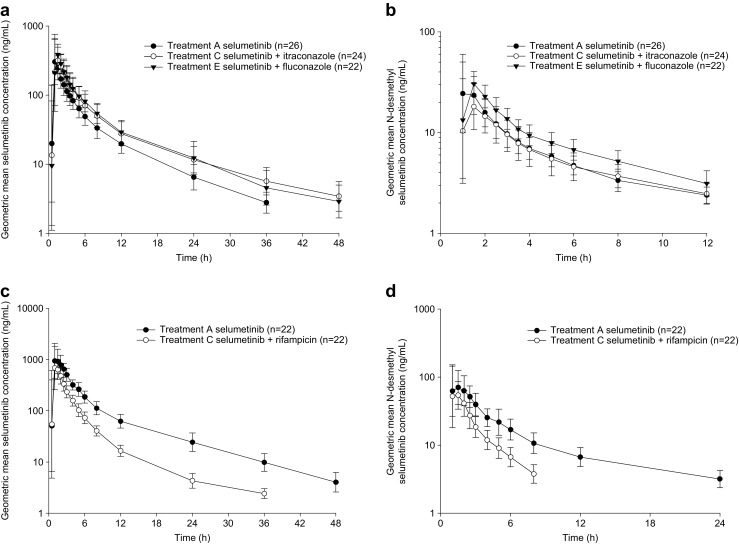
Geometric mean plasma concentrations (ng/mL) versus time of selumetinib (**a**) and *N*-desmethyl selumetinib (**b**) following single doses of selumetinib, and selumetinib co-dosed with itraconazole or fluconazole, and of selumetinib (**c**) and *N*-desmethyl selumetinib (**d**) following single doses of selumetinib, or selumetinib co-dosed with rifampicin. **a**, **b** Treatment A: 25 mg selumetinib alone on day 1, treatment C: 200 mg itraconazole twice daily on day 8 through day 11 plus 25 mg selumetinib on day 8, treatment E: 200 mg fluconazole once daily on day 8 through day 11 plus 25 mg selumetinib on day 8. **c**, **d** Treatment A: a single oral dose of 75 mg (3 × 25 mg) selumetinib administered under the fasted state on day 1, treatment C: a single daily oral dose of 600 mg rifampicin on days 12 to 14, with 75 mg selumetinib on day 12 under the fasted state

Trough plasma itraconazole concentration was well above CYP3A4 IC_50_ values [13], ranging from 595 to 776 ng/mL (geometric mean) on days 6 to 8, indicating that sufficient exposure for inhibition of CYP3A4 was achieved. Selumetinib exposure increased when co-dosed with itraconazole (Fig. 2a; Table 1). AUC was increased by approximately 49% (90% CI 40.4, 58.8), and *C*_max_ was increased by approximately 19% (90% CI 4.2, 34.9) (Table 2). Co-dosing with itraconazole also prolonged selumetinib mean terminal half-life (*t*_1/2_ ) by ~6 h and decreased apparent systemic clearance (CL/F) by approximately one third (Table 1).

**Table 1:** Summary of selumetinib and *N*-desmethyl selumetinib plasma pharmacokinetic parameters following 25 mg selumetinib co-dosing with the cytochrome P450 inhibitors, itraconazole or fluconazole (study A), and following 75 mg selumetinib co-dosing with the cytochrome P450 CYP3A4 inducer, rifampicin (study B)

	Study A			Study B	
Parameter	Selumetinib *N* = 26	Selumetinib + itraconazole *N* = 24	Selumetinib + fluconazole *N* = 22	Selumetinib *N* = 22	Selumetinib + rifampicin *N* = 22
Selumetinib					
AUC (ng h/mL) (CV%)	1180 (27.2)	1760 (31.8)	1770 (33.4)	4480 (19.9)	2200 (23.4)
*C*_max_ (ng/mL) (CV%)	409 (36.8)	484 (30.8)	509 (34.0)	1440 (36.5)	1070 (38.3)
*t*_max_ (h) median (min, max)	1.00 (0.50, 2.50)	1.01 (0.50, 2.50)	1.50 (0.50, 2.50)	1.27 (1.00, 2.50)	1.00 (0.50, 2.00)
*t*_1/2_ (h) arithmetic mean (SD)	8.24 (2.07)	14.0 (7.77)	9.75 (3.14)	9.27 (2.57)	6.69 (2.33)
CL/F (L/h) arithmetic mean (SD)	21.9 (5.76)	14.9 (4.92)	14.8 (4.39)	17.0 (3.33)	35.0 (8.46)
*N*-desmethyl selumetinib					
AUC_(0–t)_ (ng h/mL) (CV%)	83.4 (30.0)	74.6 (29.2)	117 (30.4)	404 (27.1)^a^	170 (28.4)
*C*_max_ (ng/mL) (CV%)	35.7 (37.6)	27.0 (29.4)	37.5 (33.6)	106 (41.8)	86.9 (40.4)
*t*_max_ (h) median (min, max)	1.00 (0.50, 2.50)	1.50 (1.00, 2.50)	1.50 (1.00, 2.50)	1.50 (1.00, 3.00)	1.00 (0.98, 2.50)
*t*_1/2_ (h) arithmetic mean (SD)	4.66 (2.38)	4.54 (1.77)	6.37 (3.67)	9.47 (3.24)	NR^b^
MR_AUC(0–t)_, arithmetic mean (SD)	0.0760 (0.0227)	0.0466 (0.0154)	0.0717 (0.0246)	0.0921 (0.0236)	0.0786 (0.0145)
MR_AUC_, arithmetic mean (SD)	NC	NC	NC	0.0921 (0.0236)^a^	0.0786 (0.0145)
MR_Cmax_, arithmetic mean (SD)	0.0917 (0.0312)	0.0580 (0.0175)	0.0772 (0.0254)	0.0760 (0.0187)	0.0829 (0.0163)

*AUC* area under the plasma concentration-time curve from time zero to infinity, *AUC*_*(0–t)*_ area under the plasma concentration-time curve from time zero to last quantifiable concentration, *C*_*max*_ maximum observed plasma concentration, *CL/F* apparent systemic clearance, *CV%* geometric coefficient of variation, *MR*_*AUC*_ AUC metabolite to parent ratios, *MR*_*AUC(0–t)*_ AUC_(0–t)_ metabolite to parent ratio, *MR*_*Cmax*_ *C*_max_ metabolite to parent ratio, *NC* not calculable, *NR* not reported, *SD* standard deviation, *t*_*max*_ time to *C*_max_, *t*_*1/2*_ apparent terminal half-life

^a^ *N* = 19

^b^Not reported due to insufficient detectable plasma concentration to determine a reliable half-life

**Table 2:** Statistical comparison of key pharmacokinetic exposure parameters for selumetinib and *N*-desmethyl selumetinib when co-dosed with itraconazole (*n* = 24) or fluconazole (*n* = 22), compared with selumetinib dosed alone (*n* = 26) (study A), and when co-dosed with rifampicin (*n* = 22), compared with selumetinib dosed alone (*n* = 22) (study B)

		Study A			Study B	
		Selumetinib	Selumetinib + itraconazole	Selumetinib + fluconazole	Selumetinib	Selumetinib + rifampicin
Selumetinib						
AUC (ng h/mL)	GLSMRRatio vs selumetinib (%) (90% CI)	1184	1767149.30 (140.40, 158.75)	1815153.30 (143.88, 163.34)	4482	220249.13 (45.91, 52.58)
*C*_max_ (ng/mL)	GLSMRRatio vs selumetinib (%) (90% CI)	408.6	484.5118.57 (104.20, 134.93)	512.9125.53 (109.90, 143.38)	1441	106874.07 (65.91, 83.26)
*N*-desmethyl selumetinib						
AUC_(0–t)_ (ng h/mL)	GLSMRRatio vs selumetinib (%) (90% CI)	83.39	74.0188.75 (82.03, 96.03)	116.8140.08 (129.14, 151.95)	347.3	157.445.32 (41.87, 49.06)
*C*_max_ (ng/mL)	GLSMRRatio vs selumetinib (%) (90% CI)	35.67	26.7875.09 (66.71, 84.52)	37.75105.83 (93.69, 119.56)	106.4	86.8681.62 (71.18, 93.59)

Results from a linear mixed-effects analysis of variance model using the natural logarithm of AUC, AUC_(0–t)_, and *C*_max_ as the response variables, fixed effect for treatment, and a random effect for subject

*AUC* area under the plasma concentration-time curve from time zero to infinity, *AUC*_*(0–t)*_ area under the plasma concentration-time curve from time zero to the last quantifiable concentration, *CI* confidence interval, *C*_*max*_ maximum observed plasma concentration, *GLSMR* geometric least squares mean ratio, *vs* versus

Itraconazole co-administration resulted in an initial decrease in *N*-desmethyl selumetinib concentrations compared to selumetinib alone, but beyond the 2 h post-administration, concentrations were similar (Fig. 2b). Additionally, *N*-desmethyl selumetinib AUC_(0–t)_ and *C*_max_ were decreased with co-dosing of selumetinib and itraconazole compared with selumetinib alone by 11 (90% CI 4.0, 18.0) and 25% (90% CI 15.5, 33.3), respectively (Table 2). Itraconazole decreased the mean selumetinib metabolite to parent *C*_max_ and AUC_(0–t)_ ratios (Table 1).

Compared with administration of selumetinib alone, co-dosing with itraconazole did not influence median selumetinib time to *C*_max_ (*t*_max_); median difference in *N*-desmethyl selumetinib *t*_max_ was 0.25 (0.00, 0.50 h; *P* = 0.0366). This delay was considered not clinically relevant.

#### Selumetinib co-dosed with fluconazole

Trough plasma fluconazole concentration was generally within the CYP2C19 IC_50_ values [14], ranging from 5590 to 5730 ng/mL (geometric mean) on days 6 to 8, indicating that sufficient exposure for inhibition of CYP2C19 was achieved. Selumetinib exposure was increased when co-dosed with fluconazole compared with selumetinib alone (Fig. 2a; Table 1). AUC was increased by 53% (90% CI 43.9, 63.3), and *C*_max_ was increased by 26% (90% CI 9.9, 43.4) (Table 2). Additionally, mean *t*_1/2_ of selumetinib was slightly prolonged by ~1.5 h and the mean CL/F was decreased by approximately one third (Table 1).

Fluconazole co-administration resulted in an increase in *N*-desmethyl selumetinib compared with selumetinib alone (Fig. 2b; Table 1). AUC_(0–t)_ was increased by 40% (90% CI 29.1, 52.0), but there was no effect on *C*_max_ (Table 2). Only minor differences in the metabolite to parent ratios for *C*_max_ and AUC were detected (Table 1).

Compared with administration of selumetinib alone, co-dosing with fluconazole did not impact median selumetinib *t*_max_; median difference in *N*-desmethyl selumetinib *t*_max_ was 0.25 h (0.00, 0.26 h; *P* = 0.0340). This difference was not considered clinically relevant.

#### Selumetinib co-dosed with rifampicin

Steady state rifampicin concentrations were achieved on the trial, as shown by levels similar to those reported in a previous study using similar doses of rifampicin [15] and the similar rifampicin concentrations across study days 7, 9, and 14 at 2 h post-dose (mean ± standard deviation (SD) 8820 ± 1870, 7960 ± 1660, and 7900 ± 1690 ng/mL, respectively).

The 4β-hydroxycholesterol/cholesterol ratio (a biomarker of CYP3A4 induction) was increased on days 12 (3.4-fold) and 14 (3.8-fold), following 9 and 11 days of continuous rifampicin administration, respectively, when compared to day 4 (pre-rifampicin). This increase indicated that rifampicin produced an adequate level of CYP3A4 induction to assess the effect on selumetinib exposure.

Selumetinib PK parameters following each treatment are summarized in Table 1, with statistical comparison of key PK exposure parameters summarized in Table 2. Selumetinib exposure was lower when co-dosed with rifampicin compared with selumetinib alone (Fig. 2c; Table 1). AUC was decreased by 51% (90% CI 47.4, 54.1), and *C*_max_ was reduced by 26% (90% CI 16.7, 34.1) (Table 2).

*N*-desmethyl selumetinib exposure was lower when selumetinib was co-administered with rifampicin (Fig. 2d; Table 1), with a decrease of 55% (90% CI 50.9, 58.1) in AUC_(0–t)_ and 18% (90% CI 6.4, 28.8) in *C*_max_ (Table 2). The decrease in *N*-desmethyl selumetinib plasma concentrations when co-administered with rifampicin compared with dosing alone was in proportion to the parent (Fig. 2c, d); hence, metabolite to parent mean ratios for AUC and *C*_max_ were not affected by co-dosing with rifampicin (Table 1).

Median selumetinib *t*_*max*_ was not affected by co-dosing with rifampicin (Table 1). Compared with selumetinib dosing alone, rifampicin co-administration resulted in an approximate 2.5 h shorter mean selumetinib *t*_1/2_ (9.27 ± 2.57 vs 6.69 ± 2.33 h) and a twofold increase in mean CL/F (Table 1).

*N*-desmethyl selumetinib *t*_max_ was slightly decreased when selumetinib was co-dosed with rifampicin (Table 1). *N*-desmethyl selumetinib concentrations were measurable to later time points when selumetinib was administered alone than when co-administered with rifampicin where concentrations were not quantifiable beyond 8 h post-dose. Hence, *N*-desmethyl selumetinib terminal *t*_1/2_ estimation with rifampicin co-administration was less robust and is not presented here.

### Adverse effects

No serious adverse events were reported during the itraconazole/fluconazole or the rifampicin PK studies in 48 subjects in total, and no subjects discontinued treatment due to adverse events.

In the itraconazole/fluconazole trial, 13/26 subjects (50%) reported at least one adverse event during the study. There were no major differences in incidence of adverse events reported in each treatment group. The highest number of adverse events was reported in subjects during treatment with selumetinib co-dosed with itraconazole (*n* = 7/24; 29.2%), with headache (*n* = 4/24; 16.7%) being the most commonly reported adverse event in this treatment group. Five subjects (5/24; 20.8%) reported adverse events during itraconazole-only treatment, four each during fluconazole only (4/23; 17.4%) and selumetinib co-dosed with fluconazole treatment (4/22; 18.2%), and two (2/26; 7.7%) during selumetinib-only treatment. The majority of all reported adverse events were considered mild in severity by the investigators. One subject (4.2%) reported a moderate adverse event, which is headache, during treatment with selumetinib co-dosed with itraconazole.

Overall, three subjects (11.5%) reported adverse events considered related to selumetinib treatment by the investigator; diffuse alopecia and headache, both of mild severity, were each reported in one subject (*n* = 2; 8.3%) during selumetinib and itraconazole co-dosing, and acne of mild severity was reported in one (4.5%) different subject during selumetinib co-dosed with fluconazole. Only one case of diffuse alopecia in the selumetinib co-dosed with itraconazole group and one case of vessel puncture site hemorrhage in the selumetinib co-dosed with fluconazole group, both of mild severity, were not resolved by the end of the study.

In the rifampicin trial, all 22 subjects (100%) reported at least one adverse event; all adverse events were mild in severity and considered unrelated to selumetinib treatment. Adverse events were reported in all subjects during the period of rifampicin administration (days 4–11); chromaturia was reported by all of these subjects and resolved 2 to 3 days after rifampicin treatment was ceased. Chromaturia in subjects administered rifampicin was, as expected, in line with the SPC [16]. Four subjects (18.2%) reported at least one adverse event during selumetinib-only treatment (arthralgia, *n* = 1; diarrhea, *n* = 1; increased alanine aminotransferase, *n* = 1; pruritus, *n* = 1), and one subject (4.5%) reported at least one adverse event (erythema, *n* = 1) during selumetinib co-dosed with rifampicin. All of the adverse events were considered to be mild in intensity and resolved by the end of study completion.

No clinically significant or relevant trends or changes in clinical laboratory parameters, vital signs, ECGs, ophthalmological or physical assessments were detected in either trial.

## Discussion

The drug-drug interaction (DDI) studies provide valuable knowledge into the potential treatment interactions during drug polypharmacy or concurrent medication use [17]. Such drug interactions may influence treatment safety and efficacy of the primary agent due to altered drug exposure and may provide important treatment considerations in the label.

In vitro data showed selumetinib to be mainly metabolized by CYP3A4 and CYP2C19; hence, it is important to understand whether the inhibition or induction of either of these enzymes influences selumetinib exposure in the clinic. With this in mind, a key objective for the phase I drug-drug interaction studies described was to assess the effect on the exposure of selumetinib following co-administration with either the CYP3A4 inhibitor itraconazole, the CYP2C19 inhibitor fluconazole, or the CYP3A4 inducer rifampicin, in healthy subjects. All of these agents have known potent enzyme modifying activities against these CYPs but can also modulate other CYPs. Results showed that selumetinib AUC increased by ~50% when co-administered with either itraconazole (CYP3A4 inhibitor) or fluconazole (CYP2C19 inhibitor), and that selumetinib AUC was decreased by ~50% by the CYP3A4 inducer rifampicin. These findings are in agreement with the in vitro data suggesting the involvement of these enzymes in the metabolism of selumetinib.

Inhibition of CYP3A4 by itraconazole or CYP2C19 by fluconazole affected levels of the metabolite, *N*-desmethyl selumetinib, differently. Co-administration of itraconazole resulted in a decrease in *N*-desmethyl selumetinib *C*_max_ (~25%) and AUC_0–t_ (~10%), indicating that CYP3A4 is involved in the formation of *N*-desmethyl selumetinib. This was also reflected in a decrease in the ratio of metabolite to parent for peak concentration (MR_Cmax_) and overall exposure (MR_AUC(0–t)_) when selumetinib was co-administered with itraconazole than on its own.

Co-administration of fluconazole with selumetinib did not affect *N*-desmethyl selumetinib *C*_max_, while AUC_(0–t)_ was increased, by about 40%. With the inhibition of the CYP2C19 metabolic pathway by fluconazole, more *N*-desmethyl metabolite may have formed via an alternative metabolic route. This is also reflected in the generally unchanged mean metabolite to parent exposure ratios for *C*_max_ and AUC_(0–t)_ with fluconazole co-administration, suggesting minimal involvement of CYP2C19 in the formation of *N*-desmethyl selumetinib.

A similar decrease in *N*-desmethyl selumetinib exposure to selumetinib exposure was observed when selumetinib was co-administered with rifampicin, indicating that CYP3A4 is also involved in *N*-desmethyl selumetinib metabolism. As rifampicin is also an inducer of other CYP450 enzymes to varying degrees [18, 19], including CYP2C19, the increase in selumetinib and *N*-desmethyl selumetinib metabolism may be due to a combination of both CYP3A4 and CYP2C19 inductions and potentially via other CYPs as well.

The dose of selumetinib was specifically selected to account for the potential changes in exposure on co-dosing with these potent CYP enzyme modifying agents; the resulting altered selumetinib AUC and *C*_max_ values did not exceed the safety exposure limit for healthy subjects that was derived from exposures in patients at the phase III selumetinib dose of 75 mg twice daily. No safety concerns were identified between treatment groups.

The study design of the itraconazole and fluconazole trial proved efficient, using the same control arm for both treatments with no marked complications in recruitment or subject dropout. Moreover, there were no treatment discontinuations due to any adverse event. Observed adverse events were consistent with the safety profile of selumetinib and the additional study treatments. Selumetinib was well tolerated administered as either a single dose or when co-administered with itraconazole, fluconazole, or rifampicin. There were three adverse events (alopecia, headache, and acne, which were all of mild severity) considered related to selumetinib in the itraconazole/fluconazole trial and none in the rifampicin trial.

In the PK trials discussed, results using the CYP modulator probes for inhibition of CYP3A4 (itraconazole) or CYP2C19 (fluconazole), or induction of CYP3A4 (rifampicin), are informative of the potential exposure profile for selumetinib when co-administered with other CYP3A4/CYP2C19 inhibitors and CYP3A4 inducers in the clinical setting and, thus, are useful tool probes. By administering potent CYP3A4/CYP2C19 inhibitors and CYP3A4 inducers over a prolonged, multiple-dose regimen, this likely reflects a “worst case” situation for drug-drug interactions. The results presented here are encouraging and show that changes in the PK of selumetinib are relatively small (~50%). That said, the clinical significance of these potential changes will need to be considered together with safety and efficacy data from pivotal trials to determine any advice such as caution, additional monitoring, or dose adjustment that may be appropriate when co-administration is indicated.

A potential limitation of the current studies is that selumetinib was administered as a single dose (25 or 75 mg), alone, or in combination with one other treatment in healthy subjects. In the clinical setting, patients are likely to receive one or more treatments as multiple doses. However, despite this limitation, the data collected are sufficient to provide appropriate guidance in the drug label.

Another potential limitation was the separation of the itraconazole/fluconazole dosing versus selumetinib dosing to accommodate fed/fast advice for the respective treatments. Whilst it is not ideal to have this separation, it is not expected that the somewhat reduced itraconazole/fluconazole exposure at the time the selumetinib was dosed would diminish the inhibitory effects or the measured DDI too much, and it is believed that the data give a realistic assessment of the DDI risk.

## Conclusion

Co-administration of the potent CYP3A4 inhibitor, itraconazole, or the potent CYP2C19 inhibitor, fluconazole, with selumetinib increased selumetinib exposure by ~50%. Thus, concomitant administration of potent CYP3A4 or CYP2C19 inhibitor with selumetinib may require caution. Co-administration of the potent CYP3A4 inducer, rifampicin, with selumetinib reduced exposure to selumetinib by ~50%. These study findings will be used to advise patient use in the clinical setting.

## Electronic supplementary material

Online Resource 1 (DOCX 42 kb)

Online Resource 2 (DOCX 43 kb)

Online Resource 3 (DOCX 41 kb)

Online Resource 4 (DOCX 139 kb)

## Footnotes

1 Report no. Be000021-21, data on file, AstraZeneca, Cheshire, UK
