# Supplementary material for: Effects of cytochrome P450 (CYP3A4 and CYP2C19) inhibition and induction on the exposure of selumetinib, a MEK1/2 inhibitor, in healthy subjects: results from two clinical trials
Source: Eur J Clin Pharmacol. 2016 Nov 26;73(2):175–84. doi: 10.1007/s00228-016-2153-7 (PMC5226997; doi:10.1007/s00228-016-2153-7)
Supplement: Supplementary file 1 — (DOCX 42 kb) [file 228_2016_2153_MOESM1_ESM.docx]

## Online Resource 1: Subjects

Other inclusion criteria were a creatinine clearance >50 mL/minute, as calculated using the Cockcroft-Gault formula, and eligible to receive itraconazole, fluconazole, or rifampicin in accordance with prescribing information. Although these prescribing information are targeted at the intended patient population, they were applied in the current studies to ensure that healthy subjects also met any drug-specific eligibility criteria.

Exclusion criteria for both studies included: previous randomization to and/or administration of selumetinib, current or past history of central serous retinopathy or retinal vein thrombosis, intra-ocular pressure >21 mmHg or uncontrolled glaucoma; participation in any other clinical study that included drug treatment and/or assumption of another new chemical entity within at least 30 days of the first administration of selumetinib; and any clinically significant disease or disorder that, in the opinion of the principal investigator, could put the healthy subject at risk, influence the result of the study, or influence the healthy subject’s ability to participate in the study. Preliminary data suggest that Japanese and non-Japanese Asian healthy subjects may experience higher systemic drug exposure compared to Western healthy subjects who received the same dose of selumetinib (manuscript in preparation ethnicity study). Japanese and non-Japanese Asian healthy subjects were therefore excluded from these studies.
