# Supplementary material for: Effects of cytochrome P450 (CYP3A4 and CYP2C19) inhibition and induction on the exposure of selumetinib, a MEK1/2 inhibitor, in healthy subjects: results from two clinical trials
Source: Eur J Clin Pharmacol. 2016 Nov 26;73(2):175–84. doi: 10.1007/s00228-016-2153-7 (PMC5226997; doi:10.1007/s00228-016-2153-7)
Supplement: Supplementary file 2 — (DOCX 43 kb) [file 228_2016_2153_MOESM2_ESM.docx]

## Online Resource 2: Pharmacokinetic assessments

PK variables were summarized using descriptive statistics. The PK parameters of selumetinib were derived using non-compartmental methods with Phoenix^®^-WinNonlin^®^, Professional Version 6.3. Maximum observed plasma concentration (C_max_), AUC, and AUC_(0–t)_ were assessed for statistical evaluation of effects of co-dosing with fluconazole, itraconazole, or rifampicin. An analysis of time to C_max_ (t_max_) using the Wilcoxon Signed Rank Test, and the Lehman median estimator of difference and 90% CIs was also performed in the itraconazole/fluconazole trial. Statistical analyses of PK variables were assessed according to Quintiles Standard Operating Procedures using SAS^®^ Version 9.2 or higher (SAS Institute, Inc., Cary, North Carolina, United States).

The PK analysis set includes all subjects who received a dose of selumetinib and provided evaluable PK profiles in at least one study period, without important events or protocol deviations thought to significantly affect the PK of selumetinib. Analyses were conducted for selumetinib and for the metabolite N-desmethyl selumetinib; however, the sample size was determined according to the drug interaction effect on selumetinib, rather than the metabolite.

### *Analytical methods*

Selumetinib and N-desmethyl selumetinib were analysed in plasma with K_2_EDTA anticoagulant with ranges of 2–2000 ng/ml and 2–500 ng/ml respectively. After addition of deuterated internal standard, samples were extracted by protein precipitation by the addition of 4 volumes of methanol:acetonitrile. After centrifugation, the supernatants were extracted to dryness under nitrogen at 50°C and reconstituted in methanol;water before analysis. Extraction recovery was approximately 93% for selumetinib, N-desmethyl selumetinib and internal standards. Samples were analysed within the established frozen storage (-20°C -80°C) stability periods established for selumetinib (24 months) and N-desmethyl selumetinib (9 months).

The typical liquid chromatography system was a Prominence SIL-20AC (Shimadzu, Tokyo, Japan). A Phenomenex Luna C18 column was used and maintained at 30°C. A gradient mobile phase system was used with 0.1% formic acid in water (A) or acetonitrile (B) with a flow rate of 0.500 mL/min and the gradient running from 25% B to 65% B over 2 minutes then increasing to 95% B over 0.1 minutes and held for 0.5 minutes before re-equilibration. The mass spectrometer used was an API 5000 or 5500 triple quadruple mass spectrometer (AB Sciex, Foster City, CA, USA). This was run in positive electrospray, multiple reaction monitoring, with an ionspray voltage of 5500 V and TurboIonSpray temperature of 600°C. The following typical transitions were monitored for the analytes: selumetinib 459.0/397.2; N-desmethyl selumetinib 445.3/383.1.

Precision and accuracy of the method determined during validation indicated that accuracy ranged from -10.7 to +4.0 for selumetinib and -4.5 to +12.0 for N-desmethyl selumetinib. Imprecision was ≤8.6% for selumetinib and ≤11.8% for N-desmethyl selumetinib.
