# Supplementary material for: Effects of cytochrome P450 (CYP3A4 and CYP2C19) inhibition and induction on the exposure of selumetinib, a MEK1/2 inhibitor, in healthy subjects: results from two clinical trials
Source: Eur J Clin Pharmacol. 2016 Nov 26;73(2):175–84. doi: 10.1007/s00228-016-2153-7 (PMC5226997; doi:10.1007/s00228-016-2153-7)
Supplement: Supplementary file 3 — (DOCX 41 kb) [file 228_2016_2153_MOESM3_ESM.docx]

## Online Resource 3: Safety and tolerability

The safety analysis set included any subject who received at least one dose of any of the investigational products, and in addition in the itraconazole/fluconazole trial, for whom post-dose data were available.
