# Supplementary material for: Effects of cytochrome P450 (CYP3A4 and CYP2C19) inhibition and induction on the exposure of selumetinib, a MEK1/2 inhibitor, in healthy subjects: results from two clinical trials
Source: Eur J Clin Pharmacol. 2016 Nov 26;73(2):175–84. doi: 10.1007/s00228-016-2153-7 (PMC5226997; doi:10.1007/s00228-016-2153-7)
Supplement: Supplementary file 4 — (DOCX 139 kb) [file 228_2016_2153_MOESM4_ESM.docx]

**Online Resource 4**

Flow of subjects through the itraconazole/fluconazole trial (Study A) and rifampicin trial (Study B)


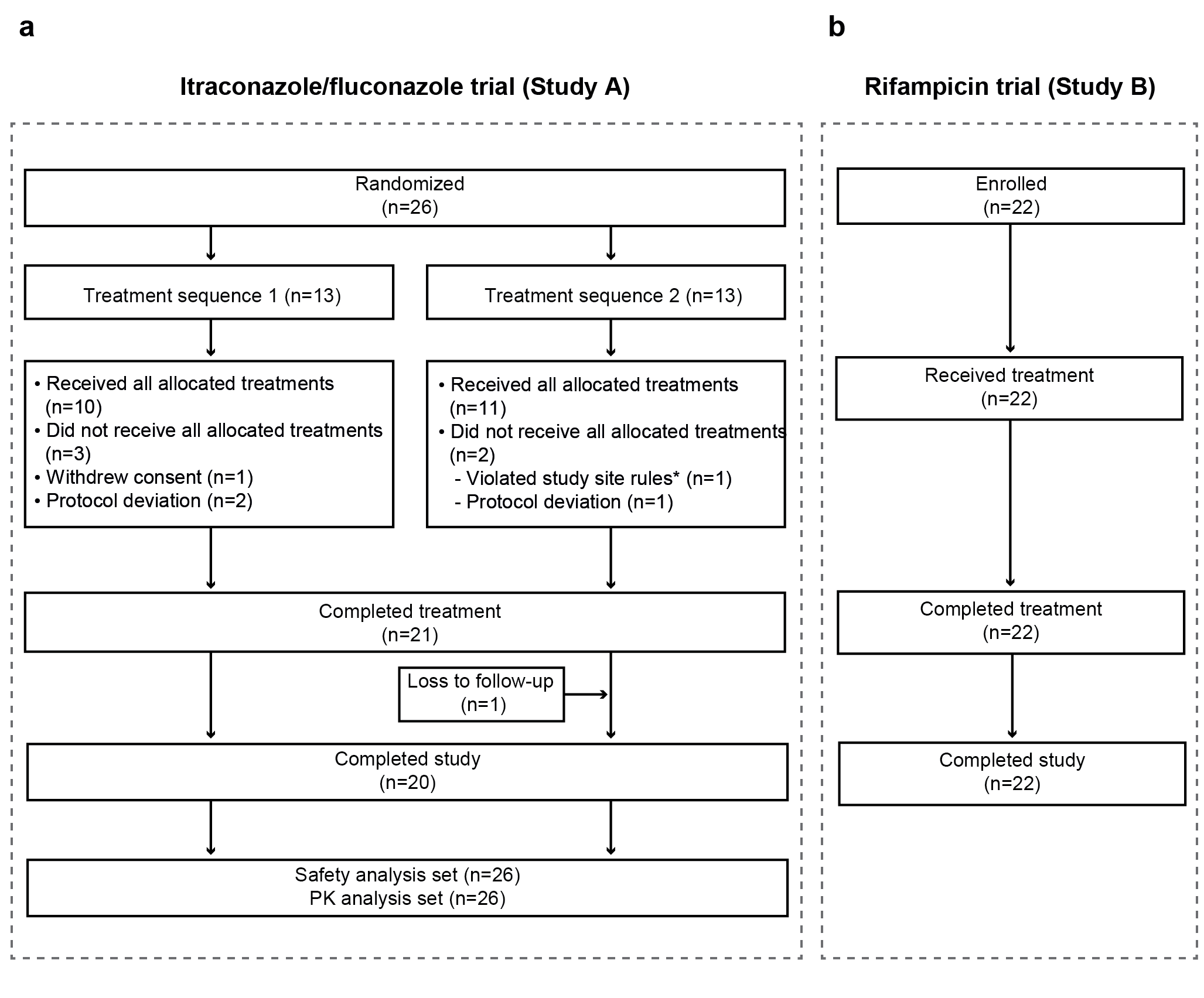


*The subject left and returned to the premises unescorted and unannounced during residential stay
